# THE IMPACT OF HOME-BASED CARE SERVICES ON ADULT CHILDREN AS CAREGIVERS OF OLDER ADULTS IN CHINA

**DOI:** 10.1093/geroni/igad104.1517

**Published:** 2023-12-21

## Abstract

The family has always been the primary resource for elder care support in China, and adult children are the main caregivers of older parents. In the background of rapid development of home-based care services for older adults, this study aims to explore the impacts of the home-based care services on the adult children as caregivers for their older parents. Based on a national representative sample of aged 60 years or older (N=8674) from the China Longitudinal Aging Social Survey (CLASS) in 2018 and 2020, this study used propensity score matching difference-in-differences method to analyze the data. Firstly, we found that home-based care services for older adults could reduce the care burden of family caregivers by sharing part of the instrumental support effectively for the elderly. Secondly, this substitution effect is more significant among the families with more dependable and vulnerable older adults. Finally, the use of home-based care services appeared not to affect the intergenerational relationships between older parents and their adult children caregivers. Home-based care services may relieve the burden of family caregivers by providing instrumental care support for older adults. Therefore, it is very necessary to accelerate the implementation of an elder care programs in community to benefit the older and younger generations in Chinese family.

